# The mediating effect of food ınsecurity in the relationship between food ınflation and eating behaviors: a case of nursing students

**DOI:** 10.3389/fpubh.2026.1943551

**Published:** 2026-09-24

**Authors:** Neşe Ataman Bor, Nesrullah Ayşin, Nuriye Nesrin İpekçi, Revşen Özer

**Affiliations:** 1 Department of Nursing, Faculty of Health Sciences, Hakkari University, Hakkari, Türkiye; 2 Health Services Vocational School, Hakkari University, Hakkari, Türkiye; 3 Yusuf Şerefoğlu Faculty of Health Sciences Nursing Department, Kilis 7 December University, Kilis, Türkiye; 4 Faculty of Health Sciences, Hakkari University, Hakkari, Türkiye

**Keywords:** food inflation, food insecurity, nursing, nutritional behavior, students

## Abstract

**Inroduction:**

Nursing students may be particularly vulnerable to food insecurity because of their intensive academic and clinical demands. Although food insecurity has been studied among university and nursing students, limited research has examined the links between food-price pressures, socioeconomic factors, and nutritional behavior, particularly the statistical mediating role of food insecurity. This study examined these relationships among nursing students.

**Methods:**

This cross-sectional correlational study included 250 undergraduate nursing students from the Department of Nursing at Kilis 7 Aralık University, Türkiye, between December 2025 and February 2026. Data were collected using a demographic information form, the Effect of Food Inflation on Nutrition Behavior Scale (EFINUB), and the Food Insecurity Experience Scale (FIES). Associations were examined using bootstrap-based path analysis, with unstandardized coefficients (B), standardized coefficients (*β*), 95% bootstrap confidence intervals (CIs), and R^2^ reported.

**Results:**

EFINUB was moderately and positively associated with FIES (*r* = 0.453, *p* < 0.001). EFINUB and FIES scores were higher among students living away from their families, reporting lower family income, prioritizing price or budget when purchasing food, and reporting that food price increases had affected their dietary habits (all p < 0.05). In the path analysis, greater food insecurity was associated with greater food inflation-related changes in nutritional behavior (*β* = 0.257, *p* < 0.001). Food insecurity statistically mediated the associations of family income, place of residence, food-purchasing priorities, and perceived effects of food price increases with EFINUB. The path model explained 44.9% of the variance in EFINUB and 21.3% of the variance in FIES.

**Discussion:**

Food insecurity may represent an important statistical pathway linking socioeconomic and food-price pressures with nutritional behavior among nursing students. Lower family income, living away from family support, and prioritizing economic considerations when purchasing food were associated with greater food insecurity and perceived changes in nutritional behavior in response to rising food prices. These findings highlight the potential relevance of university-based measures, such as affordable meal programs, campus food-support initiatives, and targeted financial assistance, to support food accessibility among nursing students experiencing food-price pressures.

## 1 Introduction

Food inflation has become an important public health and nutrition concern because rising food prices can reduce the affordability and accessibility of healthy foods, particularly among individuals with limited financial resources. In Türkiye, persistent inflation and increasing food prices have altered household purchasing decisions and increased the economic pressure associated with maintaining adequate nutrition (1). Food price increases may lead consumers to prioritize affordability over nutritional quality, reduce dietary variety, or modify meal patterns (2). At the macroeconomic level, food inflation is also associated with increased food insecurity, indicating that rising prices may affect access to sufficient and nutritious food (3). In Türkiye, food insecurity among university students has similarly been associated with poorer food consumption patterns and socioeconomic disadvantage (4, 5).

University students are particularly relevant in this context because they often experience financial constraints, changing living arrangements, and increased living costs while transitioning toward financial independence. Barriers to healthy eating among college students include food affordability, limited financial resources, time constraints, and limited access to healthier foods (2). Recent evidence also highlights food insecurity as a significant problem in higher education, with implications for students’ health and well-being (6). Thus, food insecurity among university students represents not only an individual nutritional concern but also a broader public health and health equity issue (5, 6).

Nursing students may constitute a particularly relevant subgroup because their education combines intensive theoretical coursework, laboratory learning, and compulsory clinical placements. Clinical education involves demanding schedules, patient-care responsibilities, and adaptation to healthcare environments, contributing to substantial academic and clinical stress (7–10). These demands may limit opportunities for paid employment, meal planning, and adequate food expenditure, potentially increasing vulnerability to food insecurity. Studies specifically examining nursing students support this concern. Cockerham et al. reported a substantial burden of food insecurity among baccalaureate nursing students (11), while Bydalek et al. identified financial difficulties and challenges in obtaining adequate food (12). More recent studies also indicate considerable food insecurity among undergraduate nursing students (13, 14). Therefore, nursing students should not be viewed simply as a convenient university subgroup; their distinctive educational and clinical environment may shape their access to food and nutritional behaviors.

Examining nutritional behavior in nursing students is also important beyond their individual health. Nurses play an important role in nutritional assessment, health education, disease prevention, and supporting healthy behaviors. However, evidence indicates that nursing students may have insufficient knowledge of basic, clinical, and self-care nutrition, highlighting the need to strengthen nutrition education in nursing curricula (15). Consequently, socioeconomic barriers affecting students’ own access to healthy food may intersect with their preparation for future nutrition-related care, making nutritional behavior relevant to both nursing education and clinical practice.

From a public health perspective, food inflation may influence nutritional behavior through multiple interconnected pathways. Rising food prices can increase economic pressure, which may contribute to food insecurity; food insecurity may subsequently influence food choices, dietary diversity, meal patterns, and other nutrition-related behaviors. Recent evidence suggests that food insecurity among higher education students is shaped by financial, social, and structural factors, while institutional and campus-based food-support programs may help address barriers to food access and improve support for food-insecure students (16–18). However, most existing studies have focused on the prevalence, determinants, or consequences of food insecurity among university or nursing students, rather than examining the mechanism through which food-price pressures and nutritional behavior may be linked.

In particular, limited evidence has examined whether food insecurity statistically mediates the association between perceived food-price increases and nutritional behavior among nursing students while accounting for socioeconomic and food-purchasing factors. Identifying such pathways may provide useful evidence for university food-support programs, nutrition education, student welfare policies, and nursing education strategies.

Accordingly, this study examines the relationships among perceived food-price increases, food insecurity, and nutritional behavior among undergraduate nursing students. It specifically investigates the associations of socioeconomic characteristics and food-purchasing determinants with food insecurity and nutritional behavior and evaluates whether food insecurity statistically mediates these relationships.

Research questions.

1. Is there a significant relationship between Food Price Increases on Dietary Habits (FPIDH), Food Insecurity Experience Scale (FIES), and Effect of Food Inflation on Nutrition Behavior Scale (EFINUB) among nursing students?
2. How are socioeconomic, food-purchasing, and perceptual factors associated with FIES and EFINUB among nursing students?
3. Does FIES statistically mediate the relationships between perceived food-price increases, socioeconomic factors, food-purchasing determinants, and EFINUB among nursing students?

## 2 Methods

### 2.1 Study design

This study is descriptive, cross-sectional, and relational.

### 2.2 Study setting

The study was conducted at the Faculty of Health Sciences, Department of Nursing, between December 4, 2025 and February 10, 2026. The sample consisted of nursing students enrolled in the Nursing Department of Kilis 7 December University during the specified period.

### 2.3 Population and sample

The study population consisted of 490 students enrolled in the nursing program at Kilis 7 Aralık University. The statistical power for the sample size was assessed using G*Power (version 3.1.9.7) (19). Because path analysis can be represented as a system of simultaneous multiple regression equations, the F tests test family and ‘Linear multiple regression: Fixed model, R^2^ deviation from zero’ statistical test were selected for the power analysis. The calculation was based on 17 independent variables (including dummy variables), a medium effect size (f^2^ = 0.15), a significance level of 0.05, and a statistical power of 0.95. Accordingly, the minimum required sample size was determined to be 208 participants.

In addition, representativeness was also considered using Yamane’s formula, which indicated a required sample size of 244 participants. To meet both statistical power requirements and representativeness criteria, data were collected from 250 participants. This final sample size exceeded both the minimum power-based requirement (*n* = 208) and Yamane’s suggested sample size (*n* = 244), supporting adequate statistical power and providing substantial coverage of the target population. The final sample corresponded to approximately 51% of the total population (250/490), supporting the robustness of both internal and external validity of the study findings (20).

Inclusion criteria were being enrolled as a student in the Nursing Department at Kilis 7 Aralık University, being 18 years of age or older, and providing written informed consent after being informed about the aim, procedures, and voluntary nature of the study. Exclusion criteria included students who were not willing to participate in the study, not actively attending courses during the data collection period, and those who did not complete the survey instruments fully or provided incomplete responses.

### 2.4 Data collection procedure

Data were collected between December 2025 and February 2026 using structured questionnaires. Prior to data collection, ethical approval was obtained from the relevant ethics committee, and institutional permission was granted by the Faculty of Health Sciences at Kilis 7 Aralık University. Students were informed about the purpose and scope of the study, and written informed consent was obtained from all participants. Data were collected face-to-face during class breaks without interrupting students’ classes. Participation was voluntary, and students were assured of confidentiality and their right to withdraw at any time without any academic consequences. The questionnaires were administered in classroom settings under the supervision of the researchers, and completion of the instruments took approximately 10–15 min. All collected data were anonymized and securely stored for analysis.

### 2.5 Data collection instruments

#### 2.5.1 Demographic information form

This form was developed by the researchers based on a review of the relevant literature and included questions on gender, age, place of residence, family income, employment status, and chronic disease status (21, 22).

#### 2.5.2 The effect of food inflation on nutrition behavior

The scale was developed by Durmaz et al., and its validity and reliability study was conducted by the same researchers in a Turkish sample (23). The scale consists of 36 items and is structured into four subscales based on factor analysis: food consumption (22 items), food insecurity (5 items), panic buying (6 items), and budget (3 items). The scale is a 5-point Likert-type instrument, scored as “Always” = 5, “Frequently” = 4, “Sometimes” = 3, “Rarely = 2”, and “Never” = 1. Total scores range from 36 to 180. The scale comprises four subdimensions, and each subscale can also be scored separately. There are no reverse-coded items. Higher total scores indicate a greater perceived impact of food inflation on dietary behaviors. The Cronbach’s alpha coefficient for the entire scale was reported as 0.98, while in the present study, the Cronbach’s alpha value was found to be 0.82.

Rationale for selection and conceptual role: EFINUB was selected because the primary outcome of interest in this study was not food inflation as a macroeconomic indicator, but the perceived impact of rising food prices on nutrition-related behaviors. The scale captures behavioral responses to food inflation across four domains: food consumption, food insecurity-related experiences, panic buying, and budget-related behaviors. Therefore, the total EFINUB score reflects the extent to which participants perceive that food inflation has affected their food-related behaviors and purchasing practices. In the present analytical model, EFINUB was consequently specified as the endogenous outcome variable, representing the perceived behavioral impact of food-price pressures.

#### 2.5.3 Food ınsecurity experience scale

The scale developed by Saint et al. (24) was adapted into Turkish by Kılınç et al. (25). The scale consists of 8 items in its original form and has a unidimensional structure (single-factor), meaning that it does not include sub-dimensions and assesses food insecurity experience as a single construct. The scale response format is “yes-no.” In scoring, “yes” responses are coded as 1 and “no” responses as 0. The total score ranges from 0 to 8 based on responses to the food insecurity questions. The total score is categorized into three levels: food secure (0–3), moderately food insecure (4–6), and severely food insecure (7–8). In the reliability analysis, the Cronbach’s alpha coefficient was reported as 0.85, indicating good internal consistency (25). In the present study, the Cronbach’s alpha value was found to be 0.84. Rationale for selection and conceptual role: FIES was selected because it provides a dedicated assessment of individuals’ experience of constrained or inadequate access to food due to limited resources. Unlike EFINUB, which assesses the perceived behavioral impact of food inflation, FIES focuses specifically on the experience of food insecurity as an access-related condition. Accordingly, FIES was conceptualized as an endogenous mediator in the present model, representing a potential statistical pathway through which socioeconomic and food-price-related factors may be associated with nutrition-related behavioral changes.

Conceptual distinction between EFINUB and FIES: Although EFINUB includes a subdimension labeled “food insecurity,” the two instruments were conceptually distinguished in the present study. EFINUB is a broader measure of the perceived impact of food inflation on nutrition-related and food-purchasing behaviors, whereas FIES is a dedicated measure of the experience of food insecurity resulting from constrained access to food. Thus, EFINUB was used to represent the behavioral outcome associated with food-price pressures, while FIES was used to represent food insecurity as a potential intermediary condition. The instruments were therefore not considered interchangeable measures of the same construct. Rather, the analytical model specified that socioeconomic and food-price-related conditions may be associated with food insecurity, which may in turn be statistically associated with greater perceived changes in nutrition-related behaviors captured by EFINUB.

#### 2.5.4 Data analysis

Data were analyzed using IBM SPSS Statistics (v27). Path analysis was conducted using the PATHj module in Jamovi (v2.3) and the R-based lavaan package. Parametric assumptions guided the use of Student’s t-test and one-way ANOVA for group comparisons; when assumptions were not met, the Kruskal-Wallis H test was applied. *Post-hoc* analyses were adjusted with Bonferroni correction, and effect sizes were interpreted using eta-squared. Scale scores were standardized using the POMP (Percentage of Maximum Possible) method. Path analysis assumptions were tested, maximum likelihood estimation was applied, and 5,000 bootstrap samples were used to assess indirect effects. Model evaluation considered fit indices, path coefficients, and explained variance, with significance set at *p* < 0.05.

The statistical methods used to address each research question are presented below:

1. In relation to Research Question 1 (RQ1): Pearson correlation analysis was performed to examine the bivariate relationships among perceived food price increases (FPIDH), food insecurity (FIES), and nutritional behavior (EFINUB). In addition, the direct path coefficients within the path analysis within the SEM framework framework were evaluated.
2. In relation to Research Question 2 (RQ2): Independent-samples t-tests, one-way analysis of variance (ANOVA; with Bonferroni *post-hoc* tests when the parametric assumptions were met), and the Kruskal–Wallis H test (when parametric assumptions were not met) were used to assess the associations of socioeconomic factors (family income level, place of residence, MEFP, and FPIDH) on food insecurity (FIES) and nutritional behavior (EFINUB), as well as differences between groups. Effect sizes were assessed using eta-squared (η^2^). In addition, these socioeconomic factors were included in the structural path model as exogenous predictors of FIES and EFINUB.
3. In relation to Research Question 3 (RQ3): Structural path/mediation analysis was conducted using maximum likelihood estimation and 5,000 bootstrap resamples to test the statistical mediating role of food insecurity (FIES) in the relationships between food-inflation-related determinants and nutritional behavior (EFINUB). Standardized path coefficients (β), 95% bootstrap confidence intervals (CIs), Z statistics, *p* values, and proportions of explained variance (R^2^) were reported for the direct, indirect, and total effects.

Before conducting the path analysis within the SEM framework, the underlying model assumptions were examined in detail:

- (a) Missing data analysis: Because questionnaires with incomplete responses were excluded during the data collection stage, there were no missing data in the final dataset (*n* = 250).
- (b) Outlier assessment: Univariate outliers were examined using standardized Z scores, with ±3 as the threshold, whereas multivariate outliers were assessed using Mahalanobis distance (*p* < 0.001). No extreme outliers that could adversely affect the structural model were identified.
- (c) Normality and linearity: Univariate normality was assessed using skewness and kurtosis coefficients. All values fell within the acceptable range of ±2. Linearity was confirmed through bivariate scatterplots. To minimize the potential effects of deviations from multivariate normality and to enhance the reliability of parameter estimates, maximum likelihood estimation was used together with 5,000 bootstrap resamples.
- (d) Multicollinearity: Multicollinearity among the independent variables was assessed using tolerance and variance inflation factor (VIF) values. All VIF values were below 5.0 (range = 1.12–2.05), and all tolerance values were above 0.10, confirming the absence of problematic multicollinearity among the variables.

Following these diagnostic assessments, all preliminary assumptions were considered adequately satisfied, and the path analysis within the SEM framework was subsequently performed. For all analyses, a significance level of *p* < 0.05 was considered statistically significant.

#### 2.5.5 Structural path model

To address the research questions, family income level (Income), place of residence (Residence), the most effective factor influencing food purchasing (MEFP), and the perceived impact of food price increases on nutrition (FPIDH) were included in the model as exogenous variables. The Food Insecurity Experience Scale (FIES) was specified as an endogenous mediating variable predicted by these exogenous socioeconomic, food-purchasing, and perceptual variables. The Effect of Food Inflation on Nutrition Behavior (EFINUB) was specified as the final endogenous outcome variable.

The prespecified structural path model consisted of simultaneous structural relationships representing the direct associations of the exogenous variables with FIES and EFINUB, as well as the direct association between FIES and EFINUB. Accordingly, indirect effects were examined through the pathways extending from the exogenous variables to FIES and from FIES to EFINUB. The total effect of each exogenous variable on EFINUB was also evaluated as the sum of its direct and indirect effects through FIES.

Thus, the model was specified to examine whether the associations of socioeconomic, food-purchasing, and perceptual determinants with nutritional behavior were accompanied by corresponding indirect pathways through food insecurity.

The path analysis model was specified using the following equations:

$\text{FIES}=\beta_{0}+\beta ₁\text{Income}+\beta ₂\text{Residence}+\beta ₃\text{MEFP}+\beta ₄\text{FPIDH}+\varepsilon ₁$ .

$\begin{array}{c} \text{EFINUB}=\gamma_{0}+\gamma ₁\text{Income}+\gamma ₂\text{Residence}+\gamma ₃\text{MEFP}\\ +\gamma ₄\text{FPIDH}+\gamma_{5}\text{FIES}+\varepsilon ₂ \end{array}$ .

## 3 Results

A total of 250 students participated in the study. The mean age of participants was 22.0 ± 1.8 years (range: 18–28 years). Of the participants, 60.4% were female, and the majority were living in dormitories (80.8%). Regarding socioeconomic characteristics, 61.2% of participants reported that their family income was equal to expenses. Most participants were not employed (84.8%), and 93.2% reported having no chronic disease. Price increases were identified as the most influential economic factor affecting food purchasing decisions (54.0%; Table 1).

**Table 1:** Sociodemographic characteristics of participants (*n* = 250).

Variables	Categories	n	%
Age (years)*	< 22	103	41.2
	≥ 22	147	58.8
	Mean ± SD	22.0 ± 1.8	
	Min–Max	18–28	
	Mode–Median	21–22	
Gender	Female	151	60.4
	Male	99	39.6
Family residence	Village	36	14.4
	District	78	31.2
	City center	78	31.2
	Metropolitan city	58	23.2
Current residence	Dormitory	202	80.8
	Student house	12	4.8
	Family home	36	14.4
Income level	Income less than expenses	57	22.8
	Income equal to expenses	153	61.2
	Income higher than expenses	40	16.0
Employment status	Employed	38	15.2
	Unemployed	212	84.8
Chronic disease status	Yes	17	6.8
	No	233	93.2
Most Effective Food Purchasing factor (MEFP)	Price increases	135	54.0
	Budget constraints	58	23.2
	Product availability	25	10.0
	Brand preference/quality	32	12.8
Food Price Increases on Dietary Habits (FPIDH) in the last 6 months	Yes	178	71.2
	Partly	45	18.0
	No	27	10.8

n, frequency; %, percentage; Mean ± SD, mean ± standard deviation; Min–Max, minimum–maximum; Mode–Median, most frequent value and median. *Age variable was categorized based on the median value; MEFP, Most Effective Food Purchasing factor; FPIDH, Food Price Increases on Dietary Habits.

The mean EFINUB total scale score was 113.9 ± 37.8, corresponding to 54.1% of the maximum possible score. All subdimensions of the EFINUB scale, including consumption, food security, panic buying, and budget, were slightly above the moderate level. The mean FIES score was 4.0 ± 2.8, corresponding to 50.0% of the maximum possible score. The study found that approximately half of the participants experienced moderate or high levels of food insecurity (FIES POMP ≈ 50%) and slightly above moderate levels of food inflation-related changes in nutritional behavior (EFINUB POMP ≈ 54.1%). Correlation analysis showed a moderate positive relationship between EFINUB and FIES (*r* = 0.453; *p* < 0.001; Table 2).

**Table 2:** Findings regarding scale and subscale scores, and the correlation between the two scales.

Scale/subdimension	Mean ± SD	POMP (%)	Min–Max	Mode–Median	FIES
EFINUB (Total)	113.9 ± 37.8	54.1	36–180	108–113	*r* = 0,453, *p* < 0,001
Consumption	68.5 ± 23.8	52.8	22–110	66–67.5	
Food Insecurity	16.4 ± 5.7	57.0	5–25	15–16	
Panic Buying	20.0 ± 6.6	58.3	6–30	18–20	
Budget Dimension	9.0 ± 3.7	50.0	3–15	9–9	
FIES	4.0 ± 2.8	50.0	0–8	0–4	

EFINUB, Effect of Food Inflation on Nutrition Behavior; FIES, Food Insecurity Experience Scale. Cronbach’s α, reliability coefficient; Mean ± SD, Mean ± Standard Deviation; POMP, Percentage of Maximum Possible; Min–Max, Minimum–Maximum values; Mode–Median, Most frequent value–Median value; r, Pearson Korelasyon.

Significant differences in EFINUB and FIES scores were observed according to place of residence, family income, the Most Effective Food Purchasing factor (MEFP), and the perceived effect of food price increases on dietary habits in the last six months (FPIDH; *p* < 0.05). Students living in dormitories or shared student housing had significantly higher EFINUB and FIES scores than those living with their families. Students whose income was equal to or lower than their expenses had significantly higher scores on both scales compared to those whose income exceeded their expenses. Students who reported price increases and budget constraints as the most influential factor in food purchasing had significantly higher EFINUB and FIES scores than those who prioritized product availability or brand/quality. No significant differences were found for age, gender, place of family residence, employment status, or presence of chronic disease (*p* > 0.05; Table 3).The path model was just-identified (saturated), and therefore conventional global fit indices did not provide a meaningful basis for model evaluation. The model explained 44.9% (R^2^ = 0.449) of the variance in EFINUB and 21.3% (R^2^ = 0.213) of the variance in FIES. FIES had a positive and significant effect on EFINUB (*β* = 0.257, *p* < 0.001; Table 4).

**Table 3:** Comparison of scale scores by variables.

	FIEBES			FIES		
	x̄±ss	İstatistik	*p*	x̄±ss	İstatistik	*p*
Age (Years)						
<22	112.5 ± 37.8	t:−0.466	0.642	3.9 ± 2.7	t:−0.186	0.853
≥22	114.8 ± 37.8			3.9 ± 2.8		
Gender						
Female	113.4 ± 38.2	t:−0.243	0.808	4.0 ± 2.8	t:0.381	0.704
Male	114.6 ± 37.3			3.9 ± 2.8		
Family residence						
Village	112.3 ± 40.8	F:1.58	0.195	3.7 ± 2.8	F:0.404	0.75
District	115.4 ± 39.7			3.9 ± 2.9		
City center	107.5 ± 36.5			3.9 ± 2.7		
Metropolitan	121.4 ± 34.0			4.3 ± 2.8		
Current residence						
Dorm	118.5 ± 36.6^a^	F:12.588	0.000*	4.2 ± 3.0^a^	KW:6.77	0.034*
Student house	120.2 ± 22.9^a^	a > b**		3.9 ± 0.3^a^	a > b **	
Family	85.9 ± 36.7^b^			2.7 ± 1.7^b^		
Family income level						
Income is less than expenses	127.2 ± 27.4^a^	F:26.194	0.000*	4.8 ± 2.4^a^	F:9.772	0.000*
Income equals expenses	118.1 ± 34.8^a^	a > b **		4.1 ± 2.9^a^	a > b **	
Income exceeds expenses	78.7 ± 41.5^b^			2.4 ± 2.3^b^		
Employment status						
Working	120.9 ± 36.9	t:1.244	0.215	3.6 ± 2.6	t:−0.766	0.444
Not working	112.6 ± 37.9			4.0 ± 2.8		
Chronic disease						
Yes	113.4 ± 41.3	t:−0.057	0.955	4.0 ± 2.7	t:0.073	0.942
No	113.9 ± 37.6			3.9 ± 2.8		
MEFP						
price increases	122.3 ± 31.5^a^	F:24.817	0.000*	4.3 ± 2.7^a^	F:9.506	0.000*
budget constraint	126.8 ± 29.7^a^	a > b **		4.7 ± 2.8^a^	a > b **	
Product availability	84.5 ± 36.0^b^			2.6 ± 2.7^b^		
Brand preference/quality	77.8 ± 43.6^b^			2.1 ± 2.2^b^		
FPIDH						
Yes	121 ± 34.3^a^	F:25.386	0.000*	4.2 ± 2.8^a^	F:8.503	0.000*
Partially	111.9 ± 36.8^a^	a > b **		4 ± 2.60^a^	a > b **	
No	70.2 ± 32.2^b^			1.9 ± 2.0^b^		

Mean ± SD, Mean ± Standard deviation; F, ANOVA; KW, Kruskal–Wallis test; t, Independent samples t-test; *p < 0.05; **Post-hoc comparison with Benferroni correction (no difference in similar letters); EFINUB, Effect of Food Inflation on Nutrition Behavior; FIES, Food Insecurity Experience Scale; MEFP, Most Effective Food Purchasing factor; FPIDH, Food Price Increases on Dietary Habits.

**Table 4:** Path analysis findings (model fit, direct/indirect effects, explained variance) direct effects.

Path	β	SE	LL (CI)	UL (CI)	z	*p*
Direct effects						
FIES → EFINUB	0,257	0,057	0,146	0,368	4,53	<0,001
Income *(0: Less-Equal, 1: More)* → EFINUB	−0,138	0,067	−0,269	−0,007	−2,07	0,030*
Residence *(0: Dormitory-Student house, 1: Family)* → EFINUB	−0,159	0,044	−0,245	−0,073	−3,62	<0,001
MEFP*(0: Price increases - Budget constraints, 1: Product availability - Brand preference / Quality)* → EFINUB	−0,245	0,070	−0,382	−0,108	−3,51	<0,001
FPIHD*(0: Yes - Partially, 1: No)* → EFINUB	−0,196	0,048	−0,291	−0,101	−4,06	<0,001
R^2^:0,449, %95GA(0,355, 0,538), Wald χ^2^ = 370, sd = 5, p < 0,001*						
Income *(0: Less-Equal, 1: More)* → FIES	−0,150	0,055	−0,258	−0,042	−2,71	0,010*
Residence *(0: Dormitory-Student house, 1: Family)* → FIES	−0,154	0,041	−0,235	−0,070	−3,72	<0,001*
MEFP *(0: Price increases - Budget constraints, 1: Product availability - Brand preference / Quality)* → FIES	−0,195	0,071	−0,335	−0,055	−2,73	0,010*
FPIDH *(0: Yes - Partially, 1: No)* → FIES	−0,172	0,053	−0,277	−0,067	−3,22	0,001*
R^2^:0,213, %95GA(0,128, 307], Wald χ^2^ = 105, sd = 4, p < 0,001*						
Indirect effects						
Income ⇒ FIES ⇒ EFINUB	−0,039	0,018	−0,074	−0,004	−2,189	0,029*
Residence ⇒ FIES ⇒ EFINUB	−0,039	0,012	−0,063	−0,015	−3,152	0,002*
MEFP ⇒ FIES ⇒ EFINUB	−0,050	0,022	−0,094	−0,006	−2,238	0,025*
FPIDH ⇒ FIES ⇒ EFINUB	−0,044	0,017	−0,078	−0,010	−2,536	0,011*
Model Fit Indices: *CFI:1,000; TLI:1,000; GFI:1,000; SRMR:0,000 ve RMSEA:0,000* *LUM:-1775.478; LURM:-1775.478*						

β, Standardized beta coefficient; SE, Standard error; LL, Lower limit of the 95% confidence interval (CI) for the standardized beta coefficient; UL, Upper limit of the 95% confidence interval (CI) for the standardized beta coefficient; z, Z score; EFINUB, Effect of Food Inflation on Nutrition Behavior; FIES, Food Insecurity Experience Scale; MEFP, Most Effective Food Purchasing Factor; FPIDH, Food Price Increases on Dietary Habits. R^2^, Explained variance; Wald χ^2^, Wald chi-square statistic; df, Degrees of freedom. *p < 0.05; RMSEA, Root Mean Square Error of Approximation; CFI, Comparative Fit Index; TLI, Tucker–Lewis Index; SRMR, Standardized Root Mean Square Residual; GFI, Goodness-of-Fit Index; LUM, Log-likelihood of the user model; LURM, Log-likelihood of the unrestricted model; “0” values in categorical variables represent the reference category.

Family income level, place of residence, the Most Effective Food Purchasing factor (MEFP), and the perceived effect of food price increases on dietary habits (FPIDH) over the last 6 months were identified as negative and significant predictors of EFINUB (*β* = −0.138 to −0.245; *p* < 0.05). For FIES, family income level (*β* = −0.150), place of residence (*β* = −0.154), MEFP (*β* = −0.195), and FPIDH (*β* = −0.172) were also significant negative predictors (all *p* < 0.05; Table 4).

Analyses of indirect effects showed that FIES played a partial mediating role in the relationship between all independent variables and EFINUB. The indirect effects of family income level, place of residence, MEFP, and FPIDH on EFINUB were statistically significant (*β* = −0.039 to −0.050; *p* < 0.05; Table 4).

As shown in Figure 1, the specified direct and indirect paths model consisted of two structural equations. In the first equation, food insecurity experience (FIES) was predicted by income status, place of residence, the most influential economic factor in food purchasing (MEFP), and the perceived impact of food price increases on dietary habits over the last 6 months (FPIDH). In the second equation, the effect of food inflation on nutritional behavior (EFINUB) was predicted by the same variables as well as FIES. This structure indicates that FIES functioned both as a dependent variable and a mediator, suggesting that part of the effects of the exogenous variables on EFINUB were transmitted indirectly through FIES.

**Figure 1:**
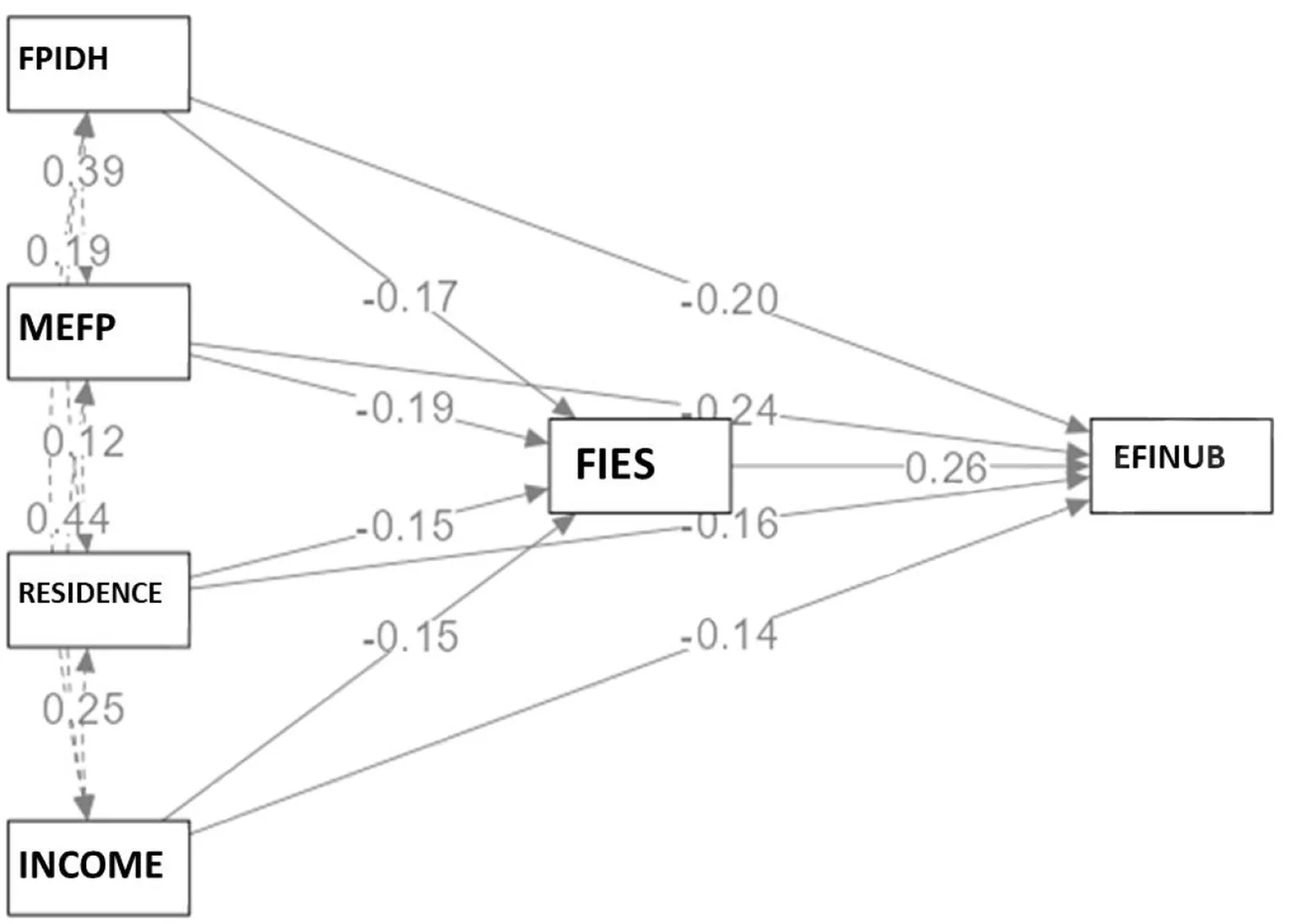
Path diagram. FPIDH, Food Price Increases on Dietary Habits (reference category: yes, partially); MEFP, Most Effective Food Purchasing Factor (References category: price increase, budget constraint). Reference category for income Less – Equal. Reference category for location Dormitory- Student house. *p < 0.05, **p < 0.01, ***p < 0.001. Numerical values represent standardized regression coefficients (β values). The coefficients presented in Table 4 are rounded in the figure.

The path diagram showed a positive relationship between FIES and EFINUB, whereas, due to the coding structure of the variables, income status, place of residence, MEFP, and FPIDH exhibited negative relationships with both FIES and EFINUB. Mediation analysis revealed that, after inclusion of FIES in the model, the effect of FPIDH on EFINUB decreased from *β* = − 0.20 to *β* = −0.17, the effect of MEFP decreased from *β* = −0.24 to *β* = −0.19, and the effect of place of residence decreased from *β* = −0.16 to *β* = −0.15, while all remained statistically significant. In contrast, the effect of income status slightly increased from *β* = −0.14 to *β* = −0.15 and also remained significant. These findings suggest that FIES plays a partial mediating role in the relationships between FPIDH, MEFP, place of residence, and EFINUB (Figure 1).

## 4 Discussion

This study addresses an important gap in the literature by examining the relationships among food inflation, food insecurity, and nutritional behaviors in nursing students using path analysis. Most previous studies have focused on general university student populations (26–28), whereas nursing students have rarely been examined as a distinct at-risk group (12). Nursing students may be particularly vulnerable because intensive academic and clinical responsibilities can limit opportunities for employment, meal planning, and regular eating patterns (29, 30). In addition, food insecurity is shaped not only by individual financial circumstances but also by environmental and institutional factors such as food affordability, accessibility, time constraints, transportation, and access to food-support programs (17, 31). Thus, nutritional difficulties among nursing students may reflect interacting economic, educational, and environmental pressures rather than individual food choices alone. As future healthcare professionals, their experiences with food affordability may also influence their understanding and communication of nutrition-related health behaviors in clinical practice (32, 33).

Approximately half of the participants experienced moderate to high food insecurity (FIES POMP ≈ 50%), while the perceived impact of food inflation on nutritional behavior was slightly above the midpoint (EFINUB POMP ≈ 54.1%). These findings may reflect the accumulation of economic pressures within a student population with limited financial resources. Financial strain may initially reduce flexibility in food purchasing and meal planning and, as resources become increasingly constrained, lead to compromises in food quantity, quality, or regularity (31, 34). This interpretation is consistent with previous studies reporting substantial food insecurity among nursing students, including prevalence estimates of approximately 43–60% (11–14). The persistence of this problem in the post-pandemic period suggests that structural economic vulnerability and the costs associated with nursing education may continue to place nursing students at risk.

Lower family income was associated with higher FIES and EFINUB scores. A plausible explanation is that when a greater proportion of income is allocated to essential expenses, students have less flexibility to absorb food-price increases without changing what, when, or how much they eat. They may substitute relatively expensive nutrient-dense foods with cheaper alternatives, reduce dietary variety, or prioritize price over nutritional value. This mechanism is particularly relevant because price increases can reduce the affordability of healthier foods, while economically constrained consumers may shift toward less expensive options (34, 35). Evidence that reducing healthy food prices increases fruit and vegetable purchases further supports the influence of food prices on dietary choices (35). Thus, the association between income and nutritional behavior may partly reflect reduced economic capacity to maintain preferred dietary practices.

The statistical mediation pattern involving food insecurity provides a possible explanation for the observed association. Limited financial resources may increase uncertainty or difficulty in obtaining sufficient food, which may subsequently alter nutritional behaviors. Previous research has similarly identified food insecurity as an important pathway linking socioeconomic disadvantage to dietary outcomes (36–40). Among university students, food insecurity has been associated with lower consumption of fruits, vegetables, and whole grains and greater consumption of fast foods, added sugars, and sugar-sweetened beverages (35). Therefore, food insecurity may function as an intermediate condition through which economic disadvantage is translated into differences in nutritional behavior. However, given the cross-sectional design, this pathway should be interpreted as a statistical mediation pattern rather than a causal process.

Students living in dormitories or student housing had higher FIES and EFINUB scores than those living with their families. This may reflect differences in both material and social food resources. Students living independently may face greater responsibility for accommodation, transportation, utilities, and food expenses, whereas those living with their families may benefit from shared household costs, prepared meals, and informal food support. Recent evidence identifies living alone as an important predictor of food insecurity among university students (31). However, residence itself should not be interpreted as a causal factor. The observed association may instead reflect the combined effects of reduced household support, greater financial responsibility, limited cooking or food-storage facilities, and restricted time for food preparation. University students also encounter multiple barriers to food access, including time constraints, transportation difficulties, and limitations in the accessibility and quality of food-support programs (17).

Students who identified price increases or budget constraints as the most influential factors in food purchasing decisions (MEFP) had higher FIES and EFINUB scores. This finding may indicate reduced economic choice in the food environment. Food purchasing decisions are shaped not only by nutrition knowledge and preferences but also by available resources, relative prices, accessibility, and convenience. When price or budget becomes the dominant consideration, students may have less capacity to prioritize nutritional quality and variety. This interpretation is consistent with evidence that food price is particularly influential among economically disadvantaged populations and that lower-cost diets may contain more energy-dense foods (34). Evidence that reducing healthy food prices increases their purchase also supports the role of economic constraints in shaping food choice (41). Previous nursing-student studies similarly indicate that students may prioritize educational and living expenses over food expenditures (11, 12).

Students who reported that food price increases had affected their dietary habits during the previous 6 months (FPIDH) also had higher FIES and EFINUB scores. Rising prices may reduce the purchasing power of a fixed student budget, prompting students to purchase cheaper foods, reduce dietary variety, modify meal frequency, or prioritize foods providing greater satiety per unit cost. Although such adaptations may maintain short-term food access, they can compromise dietary quality. Evidence among university students indicates that food insecurity is associated with poorer dietary patterns, including lower consumption of healthy foods and greater consumption of less healthy foods (35). More broadly, food prices influence food consumption, with economically disadvantaged populations generally more sensitive to price changes (34), while the affordability of healthy diets remains an important barrier among low-income populations (42). Thus, perceived food-price increases may reduce economic flexibility and increase vulnerability to food insecurity and unfavorable nutritional adaptations.

The association between food insecurity and nutritional behavior is particularly important for interpreting the path model. Food insecurity may represent a proximal condition through which socioeconomic constraints are translated into food-related behavioral changes. When access to adequate food becomes uncertain, decisions may become more strongly oriented toward affordability, satiety, and immediate availability rather than nutritional adequacy. This interpretation is consistent with evidence identifying food insecurity as a multidimensional social determinant influenced by financial, environmental, and institutional factors (17, 31, 39) and with systematic review evidence showing poorer dietary outcomes among food-insecure university students (35). The present findings therefore suggest that food insecurity may statistically connect broader socioeconomic pressures with perceived changes in nutritional behavior among nursing students.

Overall, the findings support a model in which socioeconomic constraints, living circumstances, and food-price pressures are associated with nutritional behaviors partly through food insecurity. This pattern is consistent with evidence linking low income, financial limitations, housing circumstances, and other structural conditions to food insecurity among university students (26–28, 31, 36, 37). The present study extends this literature by examining food insecurity and the perceived impact of food inflation on nutritional behavior simultaneously in nursing students. Food inflation represents an economic pressure, food insecurity reflects inadequate or uncertain access to food, and nutritional behavior may represent a behavioral response to these constraints. Nevertheless, because the study was cross-sectional and relied on self-reported measures, these relationships should be interpreted as statistical associations rather than causal pathways. Longitudinal studies are needed to determine whether food-price increases prospectively contribute to food insecurity and subsequent changes in nutritional behaviors.

From a public health perspective, these findings suggest that interventions for nursing students should extend beyond individual nutrition education. Nutrition knowledge may have limited effects when students lack the financial or environmental capacity to apply it. Recent evidence emphasizes the importance of addressing food insecurity through multiple levels, including institutional food assistance, accessible food programs, financial support, and improvements in the food environment (17, 43). Universities may therefore consider combining nutrition education with affordable healthy meals, targeted financial assistance, campus food-support programs, and early identification of students experiencing food insecurity. Such approaches may improve student well-being while also helping future nurses better understand the socioeconomic determinants of nutrition and provide realistic, context-sensitive dietary guidance to patients.

### 4.1 Limitations

Several limitations should be considered when interpreting the findings of this study. First, the cross-sectional design does not allow causal inferences to be made regarding the relationships among food inflation, food insecurity, and nutritional behaviors. Although significant associations were identified, the temporal direction of these relationships cannot be determined. In addition, the study was conducted with nursing students from a single university, which may limit the generalizability of the findings to students from other disciplines, institutions, or regions. Furthermore, the use of self-reported questionnaires may have introduced recall bias and social desirability bias, potentially affecting the accuracy of participants’ responses.

Another limitation is that, although several socioeconomic variables were included in the model, other factors that may influence food insecurity and nutritional behaviors such as household size, employment status, nutritional knowledge, mental health, and social support were not examined. Including these variables in future research may provide a more comprehensive understanding of the pathways linking food inflation, food insecurity, and dietary behaviors. Finally, because the study was conducted during a period of economic instability and rising food prices in Türkiye, the findings may reflect the specific economic conditions of that period and should therefore be interpreted within this context.

## 5 Conclusion

This study demonstrated that food insecurity statistically mediated the observed associations in the relationships between socioeconomic factors and the perceived impact of food inflation on nutritional behaviors among nursing students. Lower family income, living away from family support, prioritizing economic considerations in food purchasing decisions, and perceiving food price increases as influential on dietary habits were associated with higher levels of food insecurity and greater changes in nutritional behaviors. The findings further revealed that food insecurity partially mediated the effects of these socioeconomic determinants on nutrition related outcomes, highlighting its importance as a potential statistical pathway through which economic pressures are associated with dietary behaviors. Given that nursing students represent future healthcare professionals, addressing food insecurity and its determinants is essential not only for their personal health and academic success but also for their future capacity to promote healthy lifestyles in the populations they serve. Universities and policymakers should therefore develop targeted interventions aimed at improving food accessibility, reducing economic barriers to healthy eating, and supporting vulnerable student populations during periods of rising food prices.

From a health and nutrition policy perspective, these findings highlight the importance of incorporating food security into university health and student-support policies, particularly during periods of rising food prices. University administrations and student support services could implement subsidized or free healthy meal programs, affordable campus food options, campus food-support initiatives, and food-access assistance for economically vulnerable students. Student health and counseling services could also incorporate brief food-insecurity screening and referral pathways to available financial or food-support resources. At the policy level, higher-education authorities and policymakers could support funding mechanisms for student food assistance and policies that improve the affordability and accessibility of nutritious foods. Such strategies should give particular attention to students living away from their families and those with limited financial resources, who may have fewer informal sources of food and financial support.

The findings also have practical implications for nursing education and clinical contexts. Nursing faculty and curriculum committees could incorporate practical content on nutrition, food insecurity, and the social determinants of health into nursing curricula, while clinical educators could emphasize the recognition of food insecurity during nutritional assessment and the provision of individualized health education and appropriate referrals. However, because this cross-sectional study identifies statistical associations rather than causal relationships, these implications should be considered as directions for prevention, education, and support rather than evidence that food insecurity directly causes changes in nutritional behavior. Future longitudinal and multicenter studies should evaluate whether interventions addressing food access and economic barriers improve nutritional outcomes among nursing students and other university populations.

Future longitudinal and multicenter studies are recommended to further clarify the temporal and potentially causal pathways between food inflation, food insecurity, and nutritional behavior.

## Data Availability

The original contributions presented in the study are included in the article/supplementary material, further inquiries can be directed to the corresponding author.

## Glossary

EFINUB: Effect of Food Inflation on Nutrition Behavior
FIES: Food Insecurity Experience Scale
MEFP: Most Effective Food Purchasing Factor
FPIDH: Food Price Increases on Dietary Habits
POMP: Percentage of Maximum Possible
β: Standardized beta coefficient
SE: Standard error
LL: Lower limit of the 95% confidence interval for the standardized beta coefficient
UL: Upper limit of the 95% confidence interval for the standardized beta coefficient
z: Z score
R^2^: Explained variance
Wald χ^2^: Wald chi-square statistic
df: Degrees of freedom
RMSEA: Root Mean Square Error of Approximation
CFI: Comparative Fit Index
TLI: Tucker–Lewis Index
SRMR: Standardized Root Mean Square Residual
GFI: Goodness-of-Fit Index
LUM: Log-likelihood of the user model
LURM: Log-likelihood of the unrestricted model
